# Comparison of Fruit and Vegetable Prices between Farmers’ Markets and Supermarkets: Implications for Fruit and Vegetable Incentive Programs for Food Assistance Program Participants

**DOI:** 10.3390/nu14091842

**Published:** 2022-04-28

**Authors:** Sridharshi C. Hewawitharana, Karen L. Webb, Ron Strochlic, Wendi Gosliner

**Affiliations:** Nutrition Policy Institute, Division of Agriculture and Natural Resources, University of California, Oakland, CA 94607, USA; lkwebb@ucanr.edu (K.L.W.); rstrochlic@ucanr.edu (R.S.); wgosliner@ucanr.edu (W.G.)

**Keywords:** financial incentive, fruits and vegetables, price, farmers’ markets, SNAP

## Abstract

This cross-sectional study was part of a larger evaluation of a fruit and vegetable (FV) incentive program for Supplemental Nutrition Assistance Program (SNAP) participants in California. We examined the price differences in FV to explore whether these could help explain a previously observed lack of effect of the incentive program on FV consumption. Differences by type (organic/no-spray or conventional), among a convenience sample of farmers’ markets (*n* = 11) and nearby supermarkets (*n* = 7), were assessed using Wilcoxon rank-sum tests adjusting for clustering by market. We calculated the cost of market baskets comprising recommended FV servings for a household using median prices to consider the implications of FV price differences for SNAP shoppers who use financial incentives for FV. We found that farmers’ markets primarily offered organic FV while supermarkets primarily offered conventionally grown FV. Farmers’ market prices tended to be lower than supermarkets for organic FV but higher for conventional FV. Compared to supermarkets, the market basket composed only of organic FV cost USD 16.34 less at farmers’ markets, whereas a basket comprised of a mix of conventionally and organically grown FV cost USD 3.68 more. These differences warrant further exploration; FV price and type should be considered in studies aimed at understanding the impact of SNAP financial incentive programs.

## 1. Introduction

Poor diet quality, including low fruit and vegetable (FV) consumption, is common in the U.S. and is associated with adverse health outcomes [1,2,3,4,5]. Prior research suggests that this is especially of concern among economically disadvantaged populations, such as Supplemental Nutrition Assistance Program (SNAP) participants, as they tend to have poorer diet quality [6]. Numerous reasons have been suggested for low FV consumption among SNAP participants including: high prices and/or poor quality of FV available; distances and limited transportation to food outlets; limited time available for acquiring and preparing FV; inadequate space/equipment to prepare and store food; and poor dietary knowledge [7]. A particular challenge that SNAP participants often report is a lack of access to high-quality, affordable FV in their neighborhoods [8].

Financial incentive programs have been developed to address cost barriers with the aim of increasing FV purchasing and consumption among households with limited financial resources, particularly SNAP participants. Many such programs providing dollar-for-dollar point-of-purchase matching as an incentive for purchasing FV have been mounted in farmers’ markets throughout the U.S. [9,10,11,12,13,14,15].

While some studies have shown that these types of financial match incentive programs increase FV expenditures, or purchases, effects tend to be modest [13,16,17]. However, evidence of financial incentive programs’ impact on FV consumption is mixed [11,13,15,17,18,19,20,21,22,23,24], leaving questions about the relationship between incentives and intake. A recent national evaluation of Food Insecurity Nutrition Incentive programs found a positive impact on monthly FV expenditures among SNAP participants who shop at farmers’ markets [25], but no impact on FV consumption [26]. Similar results were found in a recent evaluation of the California Nutrition Incentive Program (CNIP), which offered a dollar-for-dollar point-of-purchase match for SNAP benefits used to purchase FV at farmers’ markets in California. This evaluation found that, while greater use of the program was associated with improved food security, reducing the odds of going hungry and eating less than they felt they should, there was no effect on FV intake [27]. 

While dollar-for-dollar point of purchase match incentive programs at farmers’ markets have not been well studied in countries outside the US, there has been some work evaluating various other financial incentives and subsidies promoting healthy food purchases internationally [28]. One study of a FV voucher program in France that provided households with vouchers for the purchase of one serving of FV per family member per day, redeemable at farmers’ markets and supermarkets, found that the program had no statistically significant effect on the proportion of adults with low intakes of FV [29]. In contrast, an evaluation of the Farmers’ Market Nutrition Coupon Program in Canada, which provided participants with weekly USD 15 coupons redeemable at farmers’ markets for the purchase of FV, dairy, eggs, nuts, meats, and fish found that 84% of survey participants reported that they ate more FV because they used the program [30]. 

These discrepancies among results could be due to many factors, such as differences in the incentive level offered, methods used to measure consumption and expenditures, or differences in sample populations. Another potential explanation may also be differences in FV prices between food retail outlets such as farmers’ markets where these financial incentives are offered and nearby supermarkets, where SNAP shoppers may otherwise shop for FV. FV price differences, if present, could potentially affect the impacts of these programs. For example, if farmers’ market FV prices are higher than supermarket prices, the incentives spent on FV at farmers’ markets may simply bridge the price difference between farmers’ markets and supermarkets, with no significant increase in FV consumption. The perception that farmers’ markets are more expensive than supermarkets has been reported to be a barrier to shopping at farmers’ markets, particularly among shoppers with low incomes [31]. However, this question has yet to be answered definitively, as studies examining price differences between farmers’ markets and supermarkets to date have had mixed results. Some find higher prices at farmers’ markets [32,33], others find lower prices [34,35,36,37,38,39,40,41], no significant differences [42,43], or mixed results, with some items more expensive at farmers’ markets and others cheaper [44,45,46]. In addition, FV prices often differ between organic and conventional FV [47]. Real and perceived price differences between organic and conventional FV have been shown to be a significant barrier to purchasing organic FV [48]. However, there is a need for further investigation of what price differences exist between organic and conventional produce in various retail settings as the current evidence is limited and mixed [38,44,45,49]. 

To address these gaps in the evidence base, this study aimed to examine (1) whether there were differences in FV prices and availability between a sample of farmers’ markets implementing point of purchase incentives compared with nearby supermarkets, overall and by FV type (organic vs. conventional) and (2) whether any observed differences in FV prices between farmers’ market and supermarkets could potentially explain the lack of effect of the program on FV consumption observed in the CNIP intervention evaluation of which this study is a part. Price differences in types of FV (conventional vs. organic) among different types of retail outlets (supermarkets vs. farmers’ markets) participating in financial incentive programs may influence the effectiveness of programs and is thus crucial for planning and tailoring programs. 

## 2. Materials and Methods

This study included a convenience sample of farmers’ markets and nearby supermarkets (study sites) throughout California that were invited to participate in an evaluation of CNIP. Farmers’ markets were first stratified by the financial incentive offered through CNIP (no incentive offered, USD 10 maximum offered, or USD 20 maximum offered) and then selected, with higher priority given to farmers’ markets with greater numbers weekly SNAP transactions, and located in a census tract with primarily English- or Spanish-speaking adults below the federal poverty line. Staff at a nonprofit organization disbursing CNIP funds to participating farmers’ markets introduced members of the research team to managers at markets identified for inclusion in the sample. The research team contacted the market managers to describe the study and request permission to collect data. All farmers’ market managers agreed to participate in the research. A local supermarket within five miles of each participating farmers’ market was invited to participate.

At each study site, data collectors documented the prices of 14 FV items during a site visit in the summer of 2018. The six fruits (cantaloupe, grapes, oranges, peaches, strawberries, watermelon) and eight vegetables (avocado, broccoli, carrots, cucumber, lettuce, onions, spinach, tomatoes) were selected based on the two-week Thrifty Food Plan [50] grocery list for a family of three. The Thrifty Food Plan is a set of market baskets constructed by the United States Department of Agriculture that represents a way in which a diet meeting age- and sex-specific dietary guidelines may be achieved with minimal cost [50]. We adapted the list of included FV items by removing items not in season in California farmers’ markets during the summer when data collection was conducted and also added a few seasonal items that were anticipated to be sold at farmers’ markets during the data collection period (strawberries, plums, avocados, grapes, and cucumber).

For each FV item, we recorded whether it was conventionally grown, organic, or not sprayed (no pesticides, herbicides, or fungicides applied). To obtain a representative median price for each item at each market, we noted the price per pound, piece, or package/bunch of each FV item at most or all stalls at each farmers’ market, up to 10 varieties (e.g., Granny Smith apples and Pink Lady apples). Similarly, we priced up to 10 varieties of each FV item at each supermarket, starting from those located closest to the store entrance and proceeding to those placed further away. Prices per piece were converted into prices per pound using standard weights from the United States Department of Agriculture Food and Nutrient Database for Dietary Studies [51]. For items sold in packages or bunches, we weighed three packages or bunches and then averaged those weights to convert the price per package or bunch to price per pound. Prices more than two standard deviations above or below the mean price were considered outliers and excluded from analyses.

To test whether FV prices were different in farmers’ markets and supermarkets, we used Wilcoxon rank sum tests adjusted for clustering by market via the Datta and Satten method, using the clusrank package in R [52]. This analysis was repeated, stratifying by FV type (conventional or organic/no-spray). Organic and no-spray FV items were combined due to limited numbers of no-spray FV items observed, and are hereafter referred to collectively as ‘organic’. 

To understand potential impacts of farmers’ market FV point-of-purchase financial incentives on participants’ purchasing power, we created hypothetical market baskets consisting of total weekly FV servings recommended by the Dietary Guidelines for Americans (DGA) for a household of three (one male and one female ages 31–50, and one 9–13 year-old boy): that is, 35 cup equivalents of fruits and 56 cup equivalents of vegetables weekly [53]. We calculated the median price across all observations of each FV item at the farmers’ markets and at supermarkets, adjusting for clustering by market, using SAS v9.4. Using these median prices, we estimated the price of a market basket comprised of equal amounts of each of the 14 FV items for which we recorded prices. We then calculated the price of the market basket for only organic FV using the median prices of those items. Cost differences of the market basket at farmers’ markets and supermarkets were then examined in relation to the most common maximum incentive amount offered at farmers’ markets in California participating in CNIP, namely USD 10.

## 3. Results

**Figure 1:**
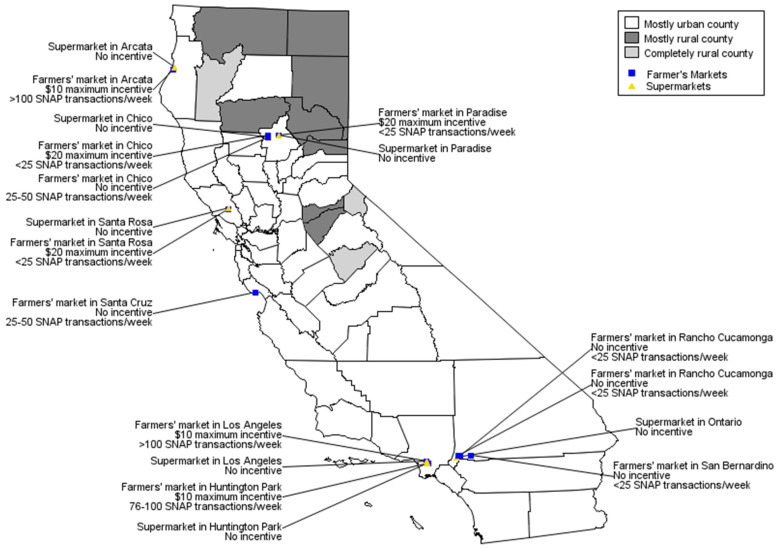
Location of farmers’ markets and supermarkets in sample, CNIP Evaluation, CA, 2018 (*n* = 11 farmers’ markets, 7 supermarkets).

Data were collected from eleven farmers’ markets and seven supermarkets across California (Figure 1). 

### 3.1. Availability of Organic and Conventionally Grown FV

Most FV items observed in supermarkets were conventionally grown (72%), whereas most items in farmers’ markets were organic (82%) (Table 1). Farmers’ markets sold few conventionally grown items and sold only organic varieties for 4 of the 14 items assessed (cantaloupe, grapes, onions, and spinach). 

### 3.2. FV Price Differences

The difference in median price overall for FV items between farmers’ markets and supermarkets (including organic and conventional) was not statistically significant. However, farmers’ market prices for conventionally grown items were significantly higher (a difference in medians of USD 1.32) than prices for comparable items in supermarkets. This significance in overall prices was driven by significantly higher prices for conventionally grown fruits at farmers’ markets (Table 1). Prices were statistically significantly higher at farmers’ markets for only a few individual items: conventionally grown strawberries (by USD 2.44), carrots over all prices (by USD 1.29) and specifically conventionally grown (by USD 1.71), and onions, over all prices (by USD 0.32). No other statistically significant differences were found in median prices between farmers’ markets and supermarkets for the remaining FV items individually, or by type—organic or conventional. Nevertheless, with the exception of three items, median prices of organic fruits and vegetables were lower at farmers’ markets compared to supermarkets.

### 3.3. Market Basket Prices

From our sample data, the cost of a hypothetical market basket for a family of three to meet the DGA for FV for one week would differ according to whether it comprised a mix of conventionally grown and organic, or only organic FV (Table 2). Assuming shoppers purchased a mix of conventionally grown and organic items proportionate to availability at the sites in this study, the basket would cost USD 61.49 at farmers’ markets and USD 57.81 at supermarkets (Table 2). However, those shoppers who purchase only organic items would pay USD 16.34 less at farmers’ markets than at supermarkets (USD 61.97 vs. USD 78.31). 

A USD 10 incentive would cover approximately 1% more of the cost of a mixed market basket at supermarkets (17.3%) than at farmers’ markets (16.3%). However, the USD 10 incentive would stretch further at farmers’ markets for a basket of organic-only FV, covering 17.3% of the cost compared with only 12.8% of the basket cost at supermarkets. 

Translated into servings of FV/person/day, the USD 10 incentive would buy an extra 0.7 or 0.75 servings/person/day from a farmers’ market or a supermarket, respectively, assuming shoppers purchased a mixed basket of conventionally grown and organic FV. For a household purchasing the organic only basket, the USD 10 incentive would buy an extra 0.7 servings of FV at a farmers’ market, compared with 0.55 servings at supermarkets.

## 4. Discussion

We found that whereas supermarkets predominantly offered conventional FV, farmers’ markets predominantly offered organic FV. Our results differ from previous findings by others who found smaller proportions of organic FV sold at farmers’ markets. Lucan et al. (2015) found that only 3.1% of fresh fruit and 7.9% of fresh vegetables sold at farmers’ markets were organic, in Bronx County, New York [32], and Claro et al. (2011) observed that 53% of selected FV items were organic at sampled farmers’ markets in Vermont [44]. By contrast, we found that a median of 82% of the selected FV items were organic at our sampled farmers’ markets in California. It is likely that the type of FV sold at farmers’ markets varies by region and community preferences. 

FV prices at farmers’ markets and supermarkets were statistically significantly different only for a few conventionally grown items. However, analyses were conducted on small numbers of observations, which may have limited our statistical power to detect differences. Our descriptive data indicate that there was a tendency for farmers’ market prices for organic items to be lower than supermarket prices. By contrast, farmers’ market prices for conventional items tended to be more expensive than supermarket prices. This study contributes to the literature on price differences between farmers’ markets and supermarkets [32,33,39,44,45,46,49,54] by highlighting the importance of considering production practices (organic versus conventional) when comparing prices. Others have also examined differences in FV prices by production practices in other parts of the United States, and while some have found similar results [38,44,45], others have seen the opposite pattern [49]. 

Similar to other studies [32,33,39,44,45,46,49,54], the magnitude of price differences we observed varied from item-to-item, with some items having no difference in median price between farmers’ markets and supermarkets and others having up to a USD 2.44 per pound difference in price. In contrast to Lucan et al. (2015), who found that for all fresh produce, any given produce item was, on average, USD 0.16 per unit of sale more expensive at farmers’ markets [32]; we found the median price per pound across all produce items assessed to be USD 0.49 per pound more at farmers’ markets. However, in both studies, this overall difference was not statistically significant.

In planning FV incentive programs, it would be useful to know the distribution of conventionally/organically grown FV items and their relative prices in different regions of the country. More research, with larger samples of farmers’ markets and supermarkets in a more diverse sample of communities across the country, is needed to gain further insight into price differences and their effects on purchasing and consumption. In addition, it would be useful to assess SNAP shopper perceived and valued benefits of purchasing organic produce, including supporting their local economies [27], protecting environmental and farmworker health [55], and reducing their exposure to pesticides [55]. Awareness of perceived and valued benefits could potentially assist in promoting incentive programs and increasing organic FV consumption.

Assuming a family of three purchased a mix of conventional and organic FV items proportional to the mix offered at retail locations, the cost of a market basket meeting the DGA recommendations was several dollars more at farmers’ markets than supermarkets. However, in contrast to Salisbury et al. (2018) [49], we found that a market basket consisting only of organic FV was much cheaper (approximately USD 16 less) at farmers’ markets than supermarkets.

These findings have implications related to the expected impact of a USD 10 financial incentive offered at farmers’ markets. For example, if a household of three that typically bought our weekly market basket with a mix of conventional and organic FV at supermarkets were to switch to buying it at a farmers’ market offering a USD 10 financial incentive, the effective amount of additional money they could spend on FV would be USD 6.32, given slightly higher median farmers’ market prices. In this case, the financial incentive would facilitate increased purchases equivalent to 0.45 servings of FV per person per day. By contrast, if this household typically purchased only organic FV and switched from shopping at a supermarket to a farmers’ market offering a USD 10 incentive, the cost of the same market basket would effectively be reduced by USD 26.34 per week. This substantial reduction in price would give this household the purchasing power to acquire an additional 1.86 servings of organic FV per person per day.

While the above situation is purely theoretical, and most price differences for individual items were not found to be statistically significant, it nevertheless illustrates how the effect of a financial incentive on a household’s ability to acquire more FV may depend on whether they purchase organic or conventional FV and where they purchase it. Studies assessing the impacts of incentive programs on FV purchases and consumption should therefore not only consider price differences but also shoppers’ typical purchasing practices when designing outcome expectations and interpreting results. Our findings may help explain the mixed evidence in the literature related to outcomes of financial incentive programs nationally. For SNAP shoppers who prefer to purchase organic FV, using dollar-for-dollar point-of-purchase financial incentive benefits at farmers’ markets may offer economic, health, and broader social benefits [56,57]. Further investigation of FV price differences in a larger sample of markets, across a larger variety of FV items, and across different seasons, days of the week, rural/urban locations, and growing climates is warranted.

Whether financial incentives for the purchase of FV are offered at supermarkets and farmers’ markets or solely at FMs remains a point for greater consideration, given the differing costs of market baskets at these venues. However, offering these incentives at farmers’ markets provides the additional benefit of supporting organic and sustainable food systems.

This study had a number of limitations. The data collected came from a small, convenience sample of markets, so non-statistically significant differences could have been due to a lack of power. Second, price observations were conducted at markets in a single state during one summer, which could limit generalizability. Third, certain conventional FV items were not sold at any of the farmers’ markets in our sample, so we were unable to calculate the price of a comparable market basket consisting only of conventional items.

Nevertheless, the study results raise an interesting point about what drives price differences in farmers’ markets and supermarket FV, and how shoppers’ established purchasing practices could factor into how they could differentially benefit from financial incentives being offered at farmers’ markets.

## Figures and Tables

**Table 1:** Produce price differences in farmers’ markets and supermarkets in California, CNIP evaluation, summer 2018.

Produce Item	Farmers’ Markets			Supermarkets			Difference in Median Prices at Farmers’ Markets and Supermarkets ^2^	*p*-Value for Difference between Farmer’s Markets and Supermarkets Prices ^1^
	Number of Markets	Number of Price Observations	Median Price per Pound (USD) (25th–75th Percentiles) ^1^	Number of Markets	Number of Price Observations	Median Price Per Pound (USD) (25th–75th Percentiles) ^1^		
Cantaloupe								
Overall	5	11	1.16 (0.75–2.16)	7	10	1.65 (0.53–2.67)	−0.49	1.000
Organic/no-spray	5	11	1.16 (0.75–2.16)	3	3	2.88 (2.47–3.41)	−1.72	0.106
Conventional	0	0	N/A ^4^	7	7	0.81 (0.27–1.72)	N/A	N/A
Grapes								
Overall	2	3	2.00 (2.00–2.38)	7	27	2.34 (1.50–2.85)	−0.34	0.512
Organic/no-spray	2	3	2.00 (2.00–2.38)	3	6	2.83 (2.42–3.24)	−0.83	0.173
Conventional	0	0	N/A	7	21	1.93 (1.48–2.69)	N/A	N/A
Oranges								
Overall	8	32	1.23 (0.99–2.05)	7	16	1.29 (0.99–1.89)	−0.06	0.973
Organic/no-spray	8	29	1.21 (0.98–1.54)	4	4	1.99 (1.25–2.00)	−0.78	0.051
Conventional	1	3	2.83 (2.75–3.00)	7	12	1.25 (0.93–1.49)	1.58	0.221
Peaches								
Overall	11	52	2.99 (2.46–3.00)	6	18	2.49 (1.49–2.87)	0.50	0.062
Organic/no-spray	7	21	3.00 (2.99–3.63)	2	4	2.99 (2.99–2.99)	0.01	0.162
Conventional	7	31	2.59 (2.29–2.80)	6	14	1.75 (1.49–2.74)	0.84	0.069
Strawberries								
Overall	11	61	3.54 (2.88–5.01)	7	19	2.87 (2.00–3.99)	0.67	0.421
Organic/no-spray	11	53	3.34 (2.75–4.77)	4	6	4.00 (3.24–4.74)	−0.66	0.208
Conventional	3	8	4.44 (3.64–5.70)	6	12	2.00 (2.00–2.99)	2.44	**0.038**
Watermelon								
Overall	7	31	0.49 (0.35–0.62)	7	16	0.50 (0.26–0.69)	−0.01	0.638
Organic/no-spray	6	30	0.48 (0.34–0.63)	2	2	1.22 (1.22–1.22)	−0.74	0.138
Conventional	1	1	0.50 (0.50–0.50)	7	14	0.44 (0.21–0.59)	0.06	0.746
Total Fruit ^3^								
Overall	11	190	2.72 (1.10–3.30)	7	106	1.97 (1.25–2.84)	0.75	0.114
Organic/no-spray	11	147	2.48 (0.83–3.35)	6	25	2.87 (2.25–3.86)	−0.39	0.215
Conventional	9	43	2.97 (2.39–2.99)	7	80	1.50 (0.87–2.50)	1.47	**0.001**
Avocado								
Overall	6	20	5.00 (3.48–5.57)	7	18	4.14 (3.24–5.81)	0.86	0.649
Organic/no-spray	6	17	5.50 (3.44–5.85)	5	6	5.62 (3.81–6.31)	−0.12	0.439
Conventional	1	3	3.81 (2.95–4.75)	7	12	3.77 (2.63–4.51)	0.04	0.809
Broccoli								
Overall	6	17	1.85 (1.49–2.34)	7	13	1.93 (1.49–2.28)	−0.08	0.246
Organic/no-spray	5	15	1.75 (1.46–2.31)	4	4	1.99 (1.91–2.69)	−0.24	0.922
Conventional	2	2	2.50 (2.50–2.50)	7	9	1.68 (1.49–2.12)	0.82	0.100
Carrots								
Overall	9	31	2.09 (1.66–2.74)	7	33	0.80 (0.52–1.48)	1.29	**0.007**
Organic/no-spray	8	27	1.99 (1.63–2.74)	6	11	1.47 (0.75–1.97)	0.52	0.331
Conventional	4	4	2.37 (2.00–2.48)	7	22	0.66 (0.50–0.96)	1.71	**0.032**
Cucumber								
Overall	10	55	1.91 (1.67–2.47)	7	20	2.07 (1.13–2.78)	−0.16	0.875
Organic/no-spray	10	46	1.96 (1.74–2.49)	3	3	2.80 (2.23–3.39)	−0.84	0.107
Conventional	3	9	1.51 (1.21–1.80)	7	17	1.88 (1.12–2.62)	−0.37	0.244
Lettuce								
Overall	10	29	2.19 (1.90–2.93)	6	17	2.45 (1.27–3.01)	−0.26	0.805
Organic/no-spray	9	27	2.21 (1.90–3.00)	5	6	2.64 (2.23–3.13)	−0.43	0.416
Conventional	1	2	2.01 (2.01–2.21)	6	11	1.94 (1.16–2.74)	0.07	0.651
Onions								
Overall	7	11	1.71 (1.38–1.94)	7	18	1.39 (0.83–1.47)	0.32	**0.006**
Organic/no-spray	7	10	1.70 (1.25–1.95)	4	4	1.49 (1.49–1.74)	0.21	0.084
Conventional	0	0	N/A	7	14	0.99 (0.79–1.43)	N/A	N/A
Spinach								
Overall	4	7	4.20 (3.85–5.30)	5	13	3.98 (2.22–6.63)	0.22	0.147
Organic/no-spray	4	7	4.20 (3.85–5.30)	3	4	5.00 (3.23–11.79)	−0.80	0.660
Conventional	0	0	N/A	5	9	3.73 (1.88–3.99)	N/A	N/A
Tomatoes								
Overall	11	56	1.99 (1.90–2.85)	7	35	2.48 (1.62–3.30)	−0.49	0.298
Organic/no-spray	11	47	1.99 (1.84–2.82)	6	13	3.64 (2.62–4.37)	−1.65	0.087
Conventional	5	9	2.00 (2.00–2.88)	7	22	1.99 (0.89–2.62)	0.01	0.063
Total Vegetable ^3^								
Overall	11	226	2.19 (1.98–2.99)	7	167	2.19 (1.13–3.34)	0.00	0.094
Organic/no-spray	11	196	2.25 (1.98–2.99)	7	51	2.94 (1.96–3.99)	−0.69	0.184
Conventional	5	29	2.01 (1.65–2.84)	7	116	1.65 (0.97–2.82)	0.36	0.061
Total Fruit and Vegetable ^3^								
Overall	11	416	2.48 (1.74–3.02)	7	273	1.99 (1.20–2.98)	0.49	0.084
Organic/no-spray	11	343	2.31 (1.51–3.22)	7	76	2.95 (2.00–3.99)	−0.64	0.139
Conventional	9	72	2.92 (2.01–2.99)	7	196	1.60 (0.96–2.74)	1.32	**0.002**

^1^ Adjusted for clustering by market; differences considered to be statistically significant when *p* < 0.05. Significant p-values shown in bold font. ^2^ Calculated as (median price of items at farmers’ markets)—(median price of items at supermarkets). ^3^ Prices for total fruit, total vegetable, and total fruit and vegetable groups include all price observations made on fruits, vegetables, and fruits and vegetables, respectively. Each price observation had equal weight in the analysis. ^4^ N/A indicates Not Applicable.

**Table 2:** Cost of meeting dietary guidelines recommended the number of fruit/vegetable cup equivalents per week for a family of three (1 woman 31–50; 1 man 31–50; 1 boy 9–13) at farmers’ markets and supermarkets.

Food Group	If Purchasing a Mix ^1^ of Conventional and Organic Produce			If Only Purchasing Organic Produce		
	Farmers’ Markets (USD)	Supermarkets (USD)	Absolute Cost Difference (USD)	Farmers’ Markets (USD)	Supermarkets (USD)	Absolute Cost Difference (USD)
Fruit	22.71	22.36	0.35	22.29	32.10	−9.81
Vegetables	38.78	35.45	3.33	39.68	46.21	−6.53
Total Fruits and Vegetables	61.49	57.81	3.68	61.97	78.31	−16.34

^1^ Mix of conventional and organic items proportionate to their availability at sites.

## Data Availability

Data not publicly available as sampled stores and markets were promised that their data would not be shared.
